# Assessment and Monitoring of the Quality of Clinical Pathways in Patients with Depressive Disorders: Results from a Multiregional Italian Investigation on Mental Health Care Quality (the QUADIM Project)

**DOI:** 10.3390/jcm12093297

**Published:** 2023-05-05

**Authors:** Matteo Monzio Compagnoni, Giulia Caggiu, Liliana Allevi, Angelo Barbato, Flavia Carle, Barbara D’Avanzo, Teresa Di Fiandra, Lucia Ferrara, Andrea Gaddini, Cristina Giordani, Michele Sanza, Alessio Saponaro, Salvatore Scondotto, Valeria D. Tozzi, Giovanni Corrao, Antonio Lora

**Affiliations:** 1 Unit of Biostatistics, Epidemiology and Public Health, Department of Statistics and Quantitative Methods, University of Milano-Bicocca, 20126 Milan, Italy; matteo.monziocompagnoni@unimib.it (M.M.C.);; 2 National Centre for Healthcare Research and Pharmacoepidemiology, University of Milano-Bicocca, 20126 Milan, Italy; 3 Department of Mental Health and Addiction Services, ASST Lecco, 23900 Lecco, Italy; 4 Department of Health Policy, Istituto di Ricerche Farmacologiche Mario Negri IRCCS, 20156 Milan, Italy; 5 Center of Epidemiology and Biostatistics, Polytechnic University of Marche, 60121 Ancona, Italy; 6 Psychologist, Previously General Directorate for Health Prevention, Ministry of Health, 00144 Rome, Italy; 7 Centre of Research on Health and Social Care Management, SDA Bocconi School of Management, Bocconi University, 20100 Milan, Italy; 8 Regional Agency for Public Health, 00198 Rome, Italy; 9 Department of Health Planning, Italian Health Ministry, 00144 Rome, Italy; 10 Department of Mental Health and Addiction Disorders Forlì-Cesena, AUSL Romagna, 48121 Cesena, Italy; 11 General Directorate of Health and Social Policies, 40127 Bologna, Italy; 12 Department of Health Services and Epidemiological Observatory, Regional Health Authority, 90145 Palermo, Italy; 13 Consultant for General Directorate for Welfare, 20124 Milan, Italy

**Keywords:** depressive disorders, mental health services, mental health care, quality of care, clinical pathways, monitoring, assessment, health care research, public health, health care utilization databases

## Abstract

Ensuring adequate quality of care to patients with severe mental disorders remains a challenge. The implementation of clinical indicators aimed at assessing the quality of health care pathways delivered is crucial for the improvement of mental health services (MHS). This study aims to evaluate the quality of care delivered to patients who are taken-into-care with depressive disorders by MHS. Thirty-four clinical indicators concerning accessibility, appropriateness, continuity, and safety were estimated using health care utilization databases from four Italian regions (Lombardy, Emilia-Romagna, Lazio, Sicily). A total of 78,924 prevalent patients treated for depressive disorders in 2015 were identified, of whom 15,234 were newly engaged by MHS. During the year of follow-up, access to psychotherapeutic interventions was low, while the intensity was adequate; 5.1% of prevalent patients received at least one hospitalization in a psychiatric ward (GHPW), and 3.3% in the cohort of newly engaged in services. Five-out-of-10 patients had contact with community services within 14 days after GHPW discharge, but less than half of patients were persistent to antidepressant drug therapy. Furthermore, prevalent patients showed an excess of mortality compared to the general population (SMR = 1.35; IC 95%: 1.26–1.44). In conclusion, the quality of health care is not delivered in accordance with evidence-based mental health standards. Evaluation of health interventions are fundamental strategies for improving the quality and equity of health care.

## 1. Introduction

Depression is a common mental health problem associated with decreased quality of life, loss of productivity, family stress, increased health care utilization and costs, and with an increase in all-cause mortality [1,2,3,4]. Moreover, not only is the prevalence of depressive disorders in the general population relatively high [5,6], but depression can also adversely affect the outcomes of chronic physical illnesses [7], including diabetes, cardiovascular diseases, cancer, and arthritis [8,9,10]. The main complications of depressive disorders include drugs, alcohol, medication abuse, family impairment, and suicidal behaviors [11,12,13]. The majority of patients with depressive disorders are treated in primary care settings, with only a small percentage of them treated in mental health services [14]. In addition, the experience of a previous episode of major depression increases the risk of experiencing a depressive relapse [15,16]. Therefore, quality improvement in the health care provided to these patients should be a priority for national health services.

Quality of care must be improved in accordance with the aim of improving the health of the population, while maintaining the goal of sustainability of the entire health care system [17]. Thus, assessment and evaluation of health interventions are fundamental strategies for improving the quality and equity of health care [18]. Quality indicators can help monitor the treatment received by people with depression in primary care, and drive needed improvements in this area [19], especially considering that the quality of health care is not always delivered in accordance with evidence-based mental health standards, and can vary widely between service providers [17,20,21,22]. This aspect is even more relevant for the mental health system currently operating at a community level. Ensuring adequate quality of care for severe mental disorders, including depression, is an important goal for research and care planning [23]. It has been difficult to compare mental health services, since there is little information on the quality provided, there is no solid evidence regarding what is meant by appropriate care, and there are no standardized measures to evaluate the quality of services provided by mental health systems [24]. Consequently, it is significantly relevant that specific indicators that are aimed at evaluating the quality of health care pathways delivered to patients with depressive disorders are implemented [25,26,27]. The indicators for assessing and monitoring health care pathways can also be applied for comparing the performance of health services and for supporting a series of clinical audits, accountability and accreditation processes, health service management, and policy guidance [28,29,30,31].

Given this background, the QUADIM project (the Italian multi-regional project “Clinical pathways in patients with severe mental disorders in Italy”) was funded by the Italian Ministry of Health (MoH) to evaluate the quality of health care pathways for patients with severe mental disorders. Specifically, in QUADIM’s framework, this study aims at assessing the quality of care routinely delivered to patients with previous and newly diagnosed depression disorders, by using a set of clinical indicators to determine the strengths and weaknesses of mental health services in four Italian regions (Lombardy, Emilia-Romagna, Lazio, and the province of Palermo, Sicily).

## 2. Materials and Methods

### 2.1. Setting

Data for the QUADIM project were retrieved retrospectively from health care utilization (HCU) databases from four Italian regions. These regions were located in the north-western (Lombardy), north-eastern (Emilia-Romagna), central (Lazio), and southern (Sicily) regions of Italy. Data collected in Sicily only comprised those from the province of Palermo; but we will refer to Sicily in the text. In Italy, mental health care is organized by local departments of mental health (DMHs) following a community-based approach. Each DMH is articulated in a complex network of territorial facilities, including community mental health centers (CMHCs), general hospital psychiatric departments (GHPWs), day-care centers (DCs), and residential facilities (CRFs). Private health care providers deliver day and residential care in partnership with the public DMHs. 

Data on psychiatric services provided by these facilities are systematically collected through the Italian ‘Mental Health Information System’ (MHIS), a national specific information system concerning psychiatric care implemented by the regional DMHs and by private facilities accredited by the NHS. Each regional MHIS collects data from all DMHs and private mental health facilities [32], allowing for comprehensive descriptions of the services’ activities, as well as the monitoring of the treatments delivered. Indeed, it also collects demographic data and ICD-10 diagnoses of all residents assisted and engaged by mental health services, as well as information related to interventions delivered to patients with a mental illness in any care setting (outpatient and home contacts, day care attendances, admissions to general hospitals, and residential facilities) [32]. The full list of interventions provided by community mental health services is reported in Supplementary Table S1. Furthermore, each Italian region implemented, in addition to the MHIS, an automated system of HCU databases to manage health services, collecting a variety of information on the national health service (NHS) beneficiaries, such as outpatient drug prescriptions, diagnosis at discharge from public or private hospitals, specialist visits, and diagnostic exams provided by the NHS. Due to a unique individual identification code used in all databases for each RHS beneficiary and automatically anonymized for privacy issues, it is possible to interconnect the regional HCU databases through a record-linkage procedure, allowing for the tracking of the complete health care pathway for each patient. More detailed information about the use of HCU databases in the mental health field can be found elsewhere [33,34,35].

As described in previous articles on the QUADIM project [35,36], HCU data were harmonized between regions, implementing consistent and comparable data extraction processes (e.g., information was encoded consistently using the same names, values, and formats). Based on a protocol previously shared and approved by the project’s working group, anonymized data were extracted and processed locally using an SAS program developed by two of the authors (M.C.M. and Caggiu G.). The diagnostic and therapeutic codes used to extract and harmonize the records and fields from the HCU databases are given in Supplementary Table S2.

### 2.2. Cohorts Selection

The target population included all NHS beneficiaries aged 18 to 65, and who were residents in the four regions involved in the QUADIM project. According to the Italian Institute of Statistics, this population amounted to 7.7 million people in 2015 (https://demo.istat.it/ accessed on 4 April 2023). All patients diagnosed with a depressive disorder, and who, from January 2015 to December 2015, were treated and received at least one contact with a DMH were identified and defined as a cohort of prevalent cases. The date of their first contact with a DMH facility during the recruitment period was recorded as the index date. To identify the cohort of patients newly engaged by mental health services, prevalent cases were excluded if they (i) had received a previous diagnosis of depression or, a diagnosis within the past two years before the index date, (ii) had been hospitalized in a psychiatric ward, and/or (iii) had received at least two consecutive antidepressant drug prescriptions [35,36]. Members of both prevalent and newly engaged cohorts accumulated person-years (PY) of follow-up from the index date through to one year after the index date (follow-up endpoint), and so were observed for at least one year.

### 2.3. Clinical Indicators

A system of clinical indicators based on evidence and best practices could help to document the quality of care, enable comparisons (benchmarking), prioritize quality improvement, support accountability, and promote transparency [29,30]. Thus, a set of quality indicators were jointly designed by two multidisciplinary expert groups, both funded by the Italian Ministry of Health (QUADIM-MAP projects, please see the Acknowledgements section) [35,37]. Starting from national recommendations [38] and international guidelines [39], as well as from the derived indicators, the interventions necessary for essential clinical pathways for the treatment and monitoring of serious mental illnesses were identified. Since the core dimensions of health quality may be articulated in sub-dimensions (i.e., accessibility, continuity, appropriateness, and safety) that could be used to evaluate the quality of care, each indicator was analyzed according to these quality dimensions. A total of 34 clinical indicators were identified, each one related to a quality-dimension of mental health care: accessibility and appropriateness (*n* = 27), continuity (*n* = 5), and safety (*n* = 2). The information contained in the regional HCU databases allowed to identify only process indicators, with the exception of mortality and general hospital psychiatric ward (GHPW) hospitalizations considered as outcome indicators [36]. More details on the rationale and process for constructing indicators to assess quality of care in severe mental disorders has been described elsewhere [34,35,36].

### 2.4. Statistical Analysis

For each region and the aggregated sample, the raw data, as well as the age- and gender-standardized prevalence and incidence rates were computed. Data from different areas were separately analyzed; the aggregated regional outputs were pooled together by using multiple meta-analysis methods to summarize the estimates.

To assess the proportion of patients who had access to specific health care interventions, and the intensity of the delivered interventions during the one-year follow-up, indicators were measured and expressed as percentages or medians. The intensity of intervention delivery was measured using the median, since several indicators had skewed distributions, and it was a more robust measure than the mean in case of outliers. The hypothesis of homogeneity among regional estimates was tested using (i) the χ^2^ test for clinical indicators expressed as proportions of treated patients, or (ii) the one-way analysis of variance (ANOVA) procedure for indicators expressed as the median number of interventions delivered per person-years of follow-up. Moreover, interregional heterogeneity of estimates was measured using the I^2^ statistic, assessing the proportion of variability explained by the differences among regions [40].

To determine the persistence with pharmacotherapy, antidepressant medications prescribed during follow-up were identified. The duration of each prescription was calculated using the defined-daily-dose method. Prescriptions were considered “consecutive” if the interval between the end of one prescription and the start of the next one was less than 90 days, and “interrupted” otherwise. Interrupted prescriptions were assumed to lead to treatment discontinuation. Similarly, all outpatient contacts provided by CMHCs or DCs were identified, and patients were considered to be persistent if they received at least one community contact every 90 days.

The expected number of deaths was calculated by grouping male and female patients by age (in 5-year age-interval groups) and multiplying the number of patients in each group by the corresponding age- and gender-specific mortality rate among the general population of each region for the year 2015 (source: Italian Institute of Statistics). The standardized mortality ratio (SMR), which gives the ratio between observed and expected deaths, was then calculated. The corresponding 95% CI were calculated by assuming that the observed number of deaths followed a Poisson distribution.

Indicators were calculated for each area and the whole sample, also according to gender. All of the differences between male and female patients were tested by calculating the standardized mean difference (SMD), which is an alternative to the *p*-value that is not influenced by the sample size. Standardized mean differences < 0.10 were considered as negligible and not statistically significant.

SAS software (version 9.4, SAS Institute, Cary, NC, USA), Excel software (from the Microsoft Office Personal Productivity Software Suite, Version 2019 16.0.6742.2048) and R software (version 4.1.3/2022, R Foundation for Statistical Computing, Vienna, Austria; packages: “metamedian”, “readxl”) were used to perform the analyses.

## 3. Results

**Figure 1:**
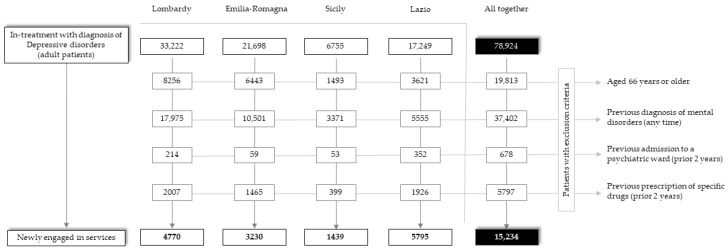
Flow-chart of the inclusion and exclusion criteria for the eligibility of patients newly admitted to services with depressive disorders in the four regions (Lombardy, Emilia-Romagna, Sicily, and Lazio), and in the whole Italian sample. QUADIM-MAP Projects, Italy, 2015–2016.

A total of 78,924 treated prevalent patients diagnosed with a depressive disorder were identified in 2015. In order to identify the cohort of patients newly engaged in services, 63,690 eligible prevalent cases were excluded (mostly because of a previous diagnosis of a depressive disorder, as shown in Figure 1), while 15,234 individuals met the inclusion criteria and were enrolled. The sociodemographic and diagnostic characteristics of the two study cohorts are shown in online Supplementary Tables S3 and S4.

Prevalence rates of treated depressive disorders per 10,000 adult inhabitants ranged between 35.1 (Lazio) and 64.7 (Sicily), with an overall age- and gender-standardized prevalence rate of 43.8. Age- and gender-standardized rates of patients newly admitted for a depressive disorder ranged between 5.7 (Lombardy) and 13.8 (Sicily) per 10,000 residents aged 18–65, with an overall incidence of 8.5. 

The estimated values of the clinical indicators for prevalent and newly admitted patients are shown in Table 1 and Table 2, respectively. Regarding the accessibility and appropriateness of territorial assistance, 92% of patients belonging to both cohorts experienced at least one outpatient contact in CMHCs or DCs. Eighty percent of prevalent patients received at least one psychiatric visit, but only 4% received a home visit. Furthermore, among prevalent patients, 38% received at least one psychosocial intervention, while 16% experienced a psychotherapy session, with a median number of five psychotherapy sessions per year. However, among patients newly admitted to services, the proportion of those who received a psychotherapy session was 22% with a median of four interventions per year. No differences between prevalent and incident patients were found for the delivery of psychosocial and psychoeducation interventions. Newly admitted patients, during the year of follow-up, spent less than half the time in residential facilities than prevalent patients.

Concerning the proportion of patients treated with antidepressant drugs among the prevalent and newly admitted cohorts, they were 65% and 43%, respectively. Only a tiny minority from both cohorts received combined drug-psychotherapy treatments. In terms of continuity, about one out of two prevalent patients received continuous community care (i.e., at least one contact with the outpatient or day care services every 90 days within the 365 days following the first contact). A similar proportion was observed for the prescription of antidepressants. The proportions of patients newly admitted to services experiencing continuity of community care and persistence of drug therapy was much lower. For prevalent cases, the continuity between inpatient and community care (i.e., the proportion of GHPW discharges followed by at least one CMHC contact within 14 days) was achieved in six individuals out of ten. Contacts in CMHCs were provided by professionals, and in four individuals out of ten, psychiatric visits were provided. Similar percentages were observed for patients newly admitted to services. Home care provided within two weeks after a hospital discharge was uncommon among prevalent patients, and even less common for newly admitted patients. Concerning the dimension of safety, the percentage of patients treated with antidepressants and who underwent an electrocardiogram and an exam for electrolytes was 20% among prevalent patients and 14% among those newly admitted to services. Mortality among prevalent patients was significantly higher than among the general population, although the difference was small (SMR = 1.13, 95% CI: 1.09–1.18). No difference was found for the newly admitted cohort: (SMR = 1.12, 95% CI: 1.00–1.25). However, among prevalent patients, men experienced a significantly higher mortality rate than the general population, whereas women had a near-zero excess mortality (males: SMR = 1.49, 95% CI: 1.40–1.58; females: SMR = 0.93, 0.88–0.99).

In both cohorts, statistically significant gender differences were observed for a very small number of mental health care interventions. The complete list of indicators stratified by gender is shown in Supplementary Tables S5 and S6.

The heterogeneity between regions was quite large for most indicators (I^2^ index often close to 100%). Compared to a prevalent patient living in Lazio, a patient living in Emilia-Romagna or Sicily received nearly half as many psychotherapy sessions. Regarding the continuity of mental health care, a patient newly admitted by MHS living in Lombardy was less likely to discontinue psychiatric care than a patient living in any of the other considered regions. High regional heterogeneity is evident in the mortality rates of the newly admitted cohort, in contrast to the prevalent cohort. Patients living in Lombardy and Emilia-Romagna have an excess mortality rate more than double that of the general population (Lombardy: SMR = 4.14, 3.41–4.99; Emilia-Romagna: SMR = 3.05, 2.32–3.96); while patients living in Lazio (SMR = 0.46, 0.37–0.58) and Sicily (SMR = 0.94, 0.68–1.28) have a lower mortality rate.

## 4. Discussion

According to the results of the QUADIM project, in Italy in 2015, people with depression had easy access to continuous community care. However, the quality of health care offered could not be considered as adequate and appropriate according to the guidelines available at that time [41], and even less adequate, when taking into account the updated guidelines recently issued [42]. Not only did the care pathways provided not appear to be consistent with the recommendations [38,39], but their heterogeneity across regions was relevant, and should be of major concern in the debate on public health [43].

More in detail, it should be noted that the rate of patients receiving the interventions that are today considered as first-line choices, such as psychotherapy, drug therapy or the combinations of both, is low, and the rate of one third for patients newly admitted to services who are not persistent with antidepressant therapy is disappointing. Although we do not have information on the quality of drug treatments, we have indirect evidence of the unsatisfactory quality of psychotherapeutic interventions, indicated by the very low number of sessions in one year. Therefore, this suggests a critical quality issue in the access to specific treatments. The relatively high number of ill-defined psychosocial interventions suggests a system based on generic supportive care. Similar results have been observed in other European countries [44]. Moreover, the treatment of depression with only drug therapy is often not enough to achieve a stable improvement in the patient’s clinical condition [45], especially when the severity of the symptoms sensibly affects the patients’ social, relational, and professional life, or when the patient expresses his/her own preference for psychological treatment [46]. According to a recent study [47], 54.5% of patients with a depressive disorder in Germany, 44.8% in France, and 38.7% in the United Kingdom, were treated with antidepressants monotherapy. In addition, the low proportions of patients treated with combined therapy is quite alarming, given the growing evidence supporting its efficacy [48,49,50].

Although the effectiveness of family and couples interventions has been proven [51,52], suggesting that treatment of depression can be improved by integrating parents, spouses, and family members into a psychosocial approach, our data show that this approach was seldom adopted. Furthermore, incentivizing the delivery of such interventions could help both patients and family members [53,54].

Generally, few patients received home care, even after a discharge from GHPW. However, few patients experienced hospital admissions and only some criteria of hospital care quality (readmission rates, length of stay) were met. Compared to acute hospital care, community residential care was more frequent and characterized by more lasting care episodes.

A significant excess in mortality was observed for the prevalent cohort (SMR = 1.13, 95% CI: 1.09–1.18), and this provides evidence that people with depression face a higher risk of all-cause death than the general population [55]. An excess mortality in individuals with depressive disorders was observed in several studies, although with rates higher than those observed in these cohorts. Patients with major depression and who were admitted to a psychiatric hospital in Taiwan between 1985 and 2008, experienced a four-fold excess of all-cause mortality (SMR equal to 3.9) [56]. Furthermore, mortality SMR was lower in Italy than in Denmark [57] and in the UK [58]. More studies are needed to better understand the reasons for the lower mortality rate in Italy in comparison with other European countries, and to investigate the heterogeneity between regions in the mortality rates of patients newly admitted to services.

Despite the fact that a large number of data have shown that the burden of depressive disorders is greater in females than in males [6,59,60], and significant gender differences have emerged in onset and in access to services and patterns of care [61], few gender differences were found in our study, with the only exception being a slightly higher number of prescriptions for antidepressant drugs among female patients than among male patients, a common finding in many surveys [62].

Finally, health, social, and cultural contexts contribute to the high variation in care pathways between regions [63]. According to several studies [63,64], the distal environment encompasses both the structure (e.g., cultural, labor market, neighborhood) and its quality (e.g., share capital). However, the critical issues, where they occur, do not specifically and exclusively concern mental health but have an impact, albeit with different methods and intensities, on all sectors of health care, being themselves symptomatic of a broader welfare system that is suffering [43]. Furthermore, according to the Global Burden of Disease Study results, depression remains among the leading causes of burden worldwide (ranked the 13th leading cause of DALYs) with prevalence estimates and disability weights comparatively higher than many other diseases [6]. In light of this, depression should be treated as one of the major public health problems and as one of the most common clinical conditions that general practitioners and specialists encounter in daily practice. 

To better understand the results obtained, it is necessary to examine the study strengths and limitations. First, this investigation is based on data from a large, unselected population. In Italy, health care is both free and universal, allowing all citizens access to essential levels of health care free of charge. Considering prevalent and newly admitted patients with depression, this study represents the largest evaluation of mental health care in Italy. This investigation is also among the most extensive surveys conducted in a European country, comparable to studies based on the Danish Depression Database [65] and other mental health registers [57]. Additionally, high-quality individual data (retrieved from the HCU databases) on outpatient and inpatient services provided by the NHS are linked to and are integrated with data on public mental health care services. Furthermore, it should be noted that information on delivered psychiatric care were retrieved from the MHIS, which is an information system regulated by law at the national level, thus permitting the collection of high-quality and complete data on regional mental health care and allowing for data comparability among regions. Thus, in a context that reflects current clinical practice, the complete care pathways of patients with severe mental disorders can be tracked and assessed, generating reliable evidence [35]. Furthermore, the present study can be reasonably defined as “population-based”, offering guarantees of representativeness and generalizability, since we were able to include all beneficiaries who were treated by public services for a given condition. Furthermore, patients newly admitted to services were identified at the time of their first contact with NHS’ mental health services, where they were diagnosed with a depressive disorder, and their full mental health care pathway was recorded.

However, several limitations affect our study. A first limitation is directly related to the data source. Based on the HCU databases, we were only able to detect the first contact with a diagnosis of depression registered in the regional public health system and occurring only in the DMHs, and this could not reflect the onset of the disorder. As reported in previous publications [35,36], although our study was designed to begin observation when a patient was diagnosed with depression, this does not always happen, possibly because we could not account for private services and primary care. It is, however, well known that the majority of depressed patients are treated in primary care, and only a small proportion of these are referred to mental health services [14].

Furthermore, as with any HCU database-based observational study, our study has the disadvantage of a lack of clinical data, such as illness severity, comorbidities, and socioeconomic status, all of which are well-known modifiers of treatment outcome and patients’ adherence. The clinical status in our cohorts can only be inferred from the diagnostic information, and information stored in HCU databases does not include clinical variables. In addition, depressive disorders are a highly heterogeneous diagnostic category, which leads to problems in the diagnosis classification and specificity of treatment. Current models classify all depressions within a single category [66]. Consequently, the lack of information on the severity of the disorder should be recognized as an important limitation of the study. Due to the lack of some information in the Italian Mental Health Information System, it was not possible to assess some quality-of-care indicators, such as coercion or case management. Furthermore, we cannot completely rule out that some differences between regions may occur because of the heterogeneity in data quality and completeness. However, it should be emphasized that, since HCU data are used to reimburse accredited and public service providers, incorrect and incomplete reporting leads to legal consequences. Finally, the validity of some estimates is based on the assumption that the prescription of a drug or the provision of a service corresponds to the consumption of the drug or the execution of the clinical control. Nonetheless, there is no guarantee that this will always be the case, and it is quite likely that prescriptions do not always result in drug consumption.

## 5. Conclusions

For assessing and monitoring the quality of health care in a community-oriented mental health system, such as the Italian one, it could be useful to define an automated and standardized set of clinical indicators. Indeed, since this evaluation system is completely based on a minimum set of HCU data already available in every Italian region, it could easily be implemented for quick and periodic evaluations. Moreover, since these measures are standardized, the treatments and care pathways provided to patients with depressive disorders can be compared both at a national and international level. As result, the quality of the mental health services delivered cannot be considered sufficient and adequate in relation to the recommendations and evidence-based findings [39].

In addition, the results of this study have shown a strong heterogeneity between the Italian regions in terms of the quality of delivered care. Since depression is a serious public health problem, increased health coverage, provision of adequate economic resources, ongoing outcome assessments and quality improvement initiatives to reduce mental health inequalities, are still a challenge to the health care systems across the world [67].

## Figures and Tables

**Table 1:** Estimation of the clinical indicators for prevalent patients diagnosed with a depressive disorder and treated by local DMHs, stratified for area (Lombardy, Emilia-Romagna, Sicily, and Lazio Regions) and for the whole sample, in the first year of follow-up. QUADIM-MAP projects, Italy, 2015–2016.

		Lombardy (*n* = 33,222)	Emilia-Romagna (*n* = 21,698)	Sicily (*n* = 6755)	Lazio (*n* = 17,249)	Whole Sample (*n* = 78,924)	I^2^ ¥
	Age- and gender-standardized treated prevalence rate (×10,000)	39.9	58.1	64.7	35.1	43.8	-
Accessibility and appropriateness of mental health care							
1	Patients with at least one outpatient contact in a CMHC or DC	90.3%	98.5%	66.2%	99.1%	92.4%	99
2	Median number of outpatient contacts in CMHCs (per PY)	4.7	7.0	5.0	6.0	5.7	99
3	Patients with at least one contact in psychiatric visit	80.5%	83.1%	60.3%	80.7%	79.5%	99
4	Median number of outpatient psychiatric visits (per PY)	3.0	3.0	2.0	4.0	3.0	99
5	Patients with at least one standardized assessment using tests	2.6%	1.5%	2.0%	4.0%	2.5%	99
6	Median number of standardized assessments using tests (per PY)	1.0	1.0	1.0	1.0	1.0	0
7	Patients with at least one home visit §	4.1%	-	5.7%	2.3%	4.4%	93
8	Median number of home visits §	3.0	-	2.0	2.0	2.3	93
9	Patients treated with at least one psychosocial intervention in a CMHC	37.8%	34.5%	38.9%	40.1%	37.5%	98
10	Median number of psychosocial interventions in CMHCs (per PY)	4.0	2.0	2.0	3.0	2.8	99
11	Patients treated with at least one psychosocial intervention in a CMHC ψ	8.8%	3.7%	2.2%	8.6%	6.8%	99
12	Median number of psychosocial interventions in CMHCs (per PY) ψ	4.0	5.0	1.0	2.0	3.0	99
13	Patients treated with at least one psychoeducation session ‡	1.7%	1.6%	3.4%	-	1.4%	95
14	Median number of psychoeducation sessions (per PY) ‡	2.0	2.0	1.0	-	1.8	83
15	Patients treated with at least one psychotherapy session	18.1%	7.4%	10.7%	22.9%	15.6%	99
16	Median number of psychotherapy sessions (per PY)	6.0	4.0	3.0	6.0	4.8	99
17	Patients with at least one outpatient carers’ contact	17.0%	20.5%	32.7%	13.6%	18.6%	99
18	Median number of interventions specifically addressed to patients’ family members (per PY)	2.0	2.0	2.0	1.0	1.8	99
19	Patients treated with antidepressants	70.1%	78.9%	51.5%	44.8%	65.4%	99
20	Patients in both psychotherapeutic-pharmacological treatment	10.9%	5.0%	6.0%	8.9%	8.4%	99
21	Patients with at least one admission in a residential facility	6.7%	6.4%	0.2%	4.4%	5.5%	99
22	Median number of days spent in residential facilities (per PY)	17.0	30.0	310.2	43.0	89.4	99
23	Patients with at least one admission in GHPW	6.2%	4.9%	4.1%	3.8%	5.1%	98
24	Median number of days spent in a GHPW (per PY)	14.0	13.0	14.0	17.0	14.4	89
25	Admissions with a length of stay in a GHPW longer than 30 days	5.9%	4.9%	1.9%	8.0%	5.7%	91
26	Unplanned re-admissions in a GHPW within seven days ¶	9.2%	6.9%	6.2%	10.7%	8.8%	75
27	Unplanned re-admissions in a GHPW within 30 days ¶	17.6%	14.6%	16.4%	18.1%	17.1%	42
Continuity of mental health care							
28	Patients with continuous community care	62.0%	39.0%	21.2%	37.2%	46.9%	99
29	Patients persistent with therapy with antidepressant	50.9%	52.7%	52.7%	48.0%	51.2%	94
30	GHPW discharges followed by any mental health outpatient contact within 14 days	50.2%	70.7%	40.7%	63.5%	55.0%	98
31	GHPW discharges followed by an outpatient psychiatric visit within 14 days	37.1%	45.9%	35.5%	49.2%	40.4%	94
32	GHPW discharges followed by home care within 14 days §	2.6%	-	0.2%	5.5%	2.9%	97
Safety of mental health care							
33	Patients monitored with electrocardiogram and exam for electrolytes (among patients treated with antidepressants)	21.8%	17.6%	21.3%	18.3%	19.8%	98
34	Mortality (SMR) and relative 95% CI	1.1 (1.0 to 1.2)	1.8 (1.1 to 1.3)	1.2 (1.1 to 1.4)	1.8 (1.1 to 1.8)	1.2 (1.1 to 1.2)	-

DMH: department of mental health. CMHC: community mental health centers; DC: day-care centers; PY: person-year; FGAs: first generation antipsychotics; SGAs: second generation antipsychotics; GHPW: general hospital psychiatric wards; SMR: standardized mortality ratio. § information for the Emilia-Romagna Region was not available for this clinical indicator, which was calculated on the 57,226 remaining patients. ψ psychosocial interventions are intended, excluding psychotherapy and psychoeducation sessions. ‡ information for the Lazio Region was not available for this clinical indicator, which was calculated on the 61,675 remaining patients. ¶ after a previous hospital admission in GHPW (statistical unit)**.** ¥ values of I^2^ for heterogeneity are percentages and can be classified as: negligible (0–25); moderate (26–50); substantive (51–75); considerable (76–100).

**Table 2:** Estimation of the clinical indicators for patients newly admitted to services with a depressive disorder and treated by local DMHs, stratified for area (Lombardy, Emilia-Romagna, Sicily, and Lazio Regions) and for the whole sample, in the first year of follow-up. QUADIM-MAP projects, Italy, 2015–2016.

		Lombardy (*n* = 4770)	Emilia-Romagna (*n* = 3230)	Sicily (*n* = 1439)	Lazio (*n* = 5795)	Whole Sample (*n* = 15,234)	I^2^ ¥
	Age- and gender-standardized treated incidence rate (×10,000)	5.7	8.7	13.8	11.8	8.5	-
Accessibility and appropriateness of mental health care							
1	Patients with at least one outpatient contact in a CMHC or DC	82.0%	98.1%	85.7%	99.2%	92.3%	99
2	Median number of outpatient contacts in CMHCs (per PY)	5.0	5.0	4.0	4.0	4.5	96
3	Patients with at least one contact in psychiatric visit	68.6%	84.2%	76.2%	74.8%	75.0%	99
4	Median number of outpatient psychiatric visits (per PY)	3.0	3.0	2.0	3.0	2.8	98
5	Patients with at least one standardized assessment using tests	4.0%	2.0%	3.0%	6.3%	4.4%	99
6	Median number of standardized assessments using tests (per PY)	1.0	1.0	1.0	1.0	1.0	0
7	Patients with at least one home visit §	1.8%	-	2.8%	1.3%	2.2%	82
8	Median number of home visits §	2.0	-	1.0	2.0	1.6	84
9	Patients treated with at least one psychosocial intervention in a CMHC	37.4%	29.1%	51.1%	36.3%	36.5%	99
10	Median number of psychosocial interventions in CMHCs (per PY)	4.0	2.0	2.0	3.0	2.7	98
11	Patients treated with at least one psychosocial intervention in a CMHC ψ	5.3%	1.1%	2.8%	5.2%	4.1%	99
12	Median number of psychosocial interventions in CMHCs (per PY) ψ	4.0	2.5	1.0	1.0	2.0	99
13	Patients treated with at least one psychoeducation session ‡	1.6%	0.6%	4.9%	-	1.1%	97
14	Median number of psychoeducation sessions (per PY) ‡	1.0	1.0	2.0	-	1.0	0
15	Patients treated with at least one psychotherapy session	25.1%	12.0%	16.7%	26.2%	22.0%	99
16	Median number of psychotherapy sessions (per PY)	5.0	4.0	3.0	4.0	4.0	89
17	Patients with at least one outpatient carers’ contact	13.2%	14.1%	41.6%	10.0%	14.9%	99
18	Median number of interventions specifically addressed to patients’ family members (per PY)	2.0	2.0	2.0	1.0	1.7	98
19	Patients treated with antidepressants	49.4%	65.4%	42.1%	26.0%	43.2%	99
20	Patients in both psychotherapeutic-pharmacological treatment	11.5%	6.0%	7.9%	6.4%	8.0%	89
21	Patients with at least one admission in a residential facility	4.3%	4.4%	0.2%	2.8%	3.4%	99
22	Median number of days spent in residential facilities (per PY)	12.0	30.0	352.2	33.0	25.7	93
23	Patients with at least one admission in a GHPW	4.3%	3.9%	2.8%	2.4%	3.3%	91
24	Median number of days spent in a GHPW (per PY)	13.0	13.0	14.0	15.0	13.6	0
25	Admissions with a length of stay in a GHPW longer than 30 days	4.4%	6.5%	3.8%	5.0%	4.9%	0
26	Unplanned re-admissions in a GHPW within seven days ¶	5.8%	7.5%	7.7%	10.0%	7.5%	0
27	Unplanned re-admissions in a GHPW within 30 days ¶	12.9%	12.9%	17.3%	16.4%	14.3%	96
Continuity of mental health care							
28	Patients with continuous community care	49.1%	13.7%	15.2%	24.8%	28.2%	99
29	Patients persistent with therapy with antidepressants	37.4%	34.4%	27.6%	31.6%	34.2%	89
30	GHPW discharges followed by any mental health outpatient contact within 14 days	57.3%	72.0%	44.2%	64.3%	60.6%	78
31	GHPW discharges followed by an outpatient psychiatric visit within 14 days	42.2%	51.6%	34.6%	48.6%	44.9%	46
32	GHPW discharges followed by home care within 14 days §	1.8%	-	1.9%	0.7%	1.4%	0
Safety of mental health care							
33	Patients monitored with electrocardiogram and exam for electrolytes (among patients treated with antidepressants)	14.0%	11.9%	17.0%	13.7%	13.6%	72
34	Mortality (SMR), and relative 95% CI	4.1 (3.4 to 5.0)	3.1 (2.3 to 4.0)	0.9 (0.7 to 1.3)	0.5 (0.4 to 0.6)	1.1 (1.0 to 1.3)	-

DMH: department of mental health. CMHC: community mental health centers; DC: day-care centers; PY: person-year; FGAs: first generation antipsychotics; SGAs: second generation antipsychotics; GHPW: general hospital psychiatric wards; SMR: standardized mortality ratio. § information for the Emilia-Romagna Region was not available for this clinical indicator, which was calculated on the 12,004 remaining patients. ψ psychosocial interventions are intended, excluding psychotherapy and psychoeducation sessions. ‡ information for the Lazio Region was not available for this clinical indicator, which was calculated on the 9439 remaining patients. ¶ after a previous hospital admission in GHPW (statistical unit)**.** ¥ values of I^2^ for heterogeneity are percentages and can be classified as: negligible (0–25); moderate (26–50); substantive (51–75); considerable (76–100).

## Data Availability

The data that support the findings of this study are available from the regions of Lombardy, Lazio, and Emilia-Romagna, and the Province of Palermo, but restrictions apply on the availability of these data, which were used under license for the current study, and so are not publicly available. Data are however available from the authors upon reasonable request and with permission from the regions involved in this study.

## Supplementary Materials

The following supporting information can be downloaded at: https://www.mdpi.com/article/10.3390/jcm12093297/s1, Table S1: Service interventions, treatments and activities delivered by community mental health centers (CMHCs) and day centers (DCs), and their classification in the Italian Mental Health Information System; Table S2: Diagnostic and therapeutic (ICD-9-CM, ICD-10, and ATC) codes used in the current study for drawing records and fields from health care utilization databases; Table S3: Baseline characteristics of prevalent patients with depressive disorder treated by DMHs in four Italian regions (Lombardy, Emilia Romagna, Sicily, and Lazio) and in the whole sample. Italy, QUADIM-MAP projects, Italy, 2015–2016; Table S4: Baseline characteristics of patients newly admitted to services with a depressive disorder and treated by DMHs in the four Italian areas (Lombardy, Emilia, Sicily, and Lazio Regions) and in the whole sample. Italy, QUADIM-MAP projects, Italy, 2015–2016; Table S5: Clinical indicators estimated from the whole sample and according to gender, in the first year of follow-up for prevalent patients with a depressive disorder treated by DMHs in the four Italian regions (Lombardy, Emilia Romagna, Sicily, and Lazio). QUADIM-MAP projects, Italy, 2015–2016; Table S6: Estimation of the clinical indicators for the whole sample and according to gender, in the first year of follow-up for patients newly admitted to services with a depressive disorder and treated by DMHs in the four Italian regions (Lombardy, Emilia Romagna, Sicily, and Lazio). QUADIM-MAP projects, Italy, 2015–2016; Acknowledgments: to the “QUADIM project” and “Monitoring and assessing diagnostic-therapeutic paths (MAP)” working groups of the Italian Ministry of Health.

## References

[1] Blazer D.G. (2003). Depression in Late Life: Review and Commentary. J. Gerontol. Ser. A.

[2] Blazer D.G., Hybels C.F., Pieper C.F. (2001). The Association of Depression and Mortality in Elderly Persons: A Case for Multiple, Independent Pathways. J. Gerontol. Ser. A.

[3] IsHak W.W., Greenberg J.M., Balayan K., Kapitanski N., Jeffrey J., Fathy H., Fakhry H., Rapaport M.H. (2011). Quality of Life: The Ultimate Outcome Measure of Interventions in Major Depressive Disorder. Harv. Rev. Psychiatry.

[4] Papakostas G.I., Petersen T., Mahal Y., Mischoulon D., Nierenberg A.A., Fava M. (2004). Quality of Life Assessments in Major Depressive Disorder: A Review of the Literature. Gen. Hosp. Psychiatry.

[5] McCarron R.M., Shapiro B., Rawles J., Luo J. (2021). Depression. Ann. Intern. Med..

[6] GBD 2019 Mental Disorders Collaborators (2022). Global, Regional, and National Burden of 12 Mental Disorders in 204 Countries and Territories, 1990–2019: A Systematic Analysis for the Global Burden of Disease Study 2019. Lancet Psychiatry.

[7] Corrao G., Monzio Compagnoni M., Valsassina V., Lora A. (2021). Assessing the Physical Healthcare Gap among Patients with Severe Mental Illness: Large Real-World Investigation from Italy. BJPsych Open.

[8] Barnett K., Mercer S.W., Norbury M., Watt G., Wyke S., Guthrie B. (2012). Epidemiology of Multimorbidity and Implications for Health Care, Research, and Medical Education: A Cross-Sectional Study. Lancet.

[9] Moussavi S., Chatterji S., Verdes E., Tandon A., Patel V., Ustun B. (2007). Depression, Chronic Diseases, and Decrements in Health: Results from the World Health Surveys. Lancet Lond. Engl..

[10] Saxena S., Maj M. (2017). Physical Health of People with Severe Mental Disorders: Leave No One Behind. World Psychiatry.

[11] Levola J., Pitkänen T., Kampman O., Aalto M. (2018). The Association of Alcohol Use and Quality of Life in Depressed and Non-Depressed Individuals: A Cross-Sectional General Population Study. Qual. Life Res..

[12] Miret M., Ayuso-Mateos J.L., Sanchez-Moreno J., Vieta E. (2013). Depressive Disorders and Suicide: Epidemiology, Risk Factors, and Burden. Neurosci. Biobehav. Rev..

[13] Wang J., Wu X., Lai W., Long E., Zhang X., Li W., Zhu Y., Chen C., Zhong X., Liu Z. (2017). Prevalence of Depression and Depressive Symptoms among Outpatients: A Systematic Review and Meta-Analysis. BMJ Open.

[14] Cuijpers P., Quero S., Dowrick C., Arroll B. (2019). Psychological Treatment of Depression in Primary Care: Recent Developments. Curr. Psychiatry Rep..

[15] Agosti V. (2014). Predictors of Remission from Chronic Depression: A Prospective Study in a Nationally Representative Sample. Compr. Psychiatry.

[16] Markkula N., Härkänen T., Nieminen T., Peña S., Mattila A.K., Koskinen S., Saarni S.I., Suvisaari J. (2016). Prognosis of Depressive Disorders in the General Population– Results from the Longitudinal Finnish Health 2011 Study. J. Affect. Disord..

[17] Berwick D.M., Nolan T.W., Whittington J. (2008). The Triple Aim: Care, Health, and Cost. Health Aff. Proj. Hope.

[18] Agabiti N., Davoli M., Fusco D., Stafoggia M., Perucci C.A. (2011). Comparative Outcome Evaluation for Health Interventions. Epidemiol. Prev..

[19] Duhoux A., Fournier L., Menear M. (2011). Quality Indicators for Depression Treatment in Primary Care: A Systematic Literature Review. Curr. Psychiatry Rev..

[20] Institute of Medicine (US) Committee (2006). Crossing the Quality Chasm: Adaptation to Mental Health and Addictive Disorders. Improving the Quality of Health Care for Mental and Substance-Use Conditions: Quality Chasm Series.

[21] Lora A., Conti V., Leoni O., Rivolta A.L. (2011). Adequacy of Treatment for Patients with Schizophrenia Spectrum Disorders and Affective Disorders in Lombardy, Italy. Psychiatr. Serv..

[22] McGlynn E.A., Asch S.M., Adams J., Keesey J., Hicks J., DeCristofaro A., Kerr E.A. (2003). The Quality of Health Care Delivered to Adults in the United States. N. Engl. J. Med..

[23] Malhi G.S., Mann J.J. (2018). Depression. Lancet.

[24] Kilbourne A.M., Keyser D., Pincus H.A. (2010). Challenges and Opportunities in Measuring the Quality of Mental Health Care. Can. J. Psychiatry.

[25] OECD (2021). A New Benchmark for Mental Health Systems: Tackling the Social and Economic Costs of Mental Ill-Health.

[26] Rotar A.M., van den Berg M.J., Kringos D.S., Klazinga N.S. (2016). Reporting and Use of the OECD Health Care Quality Indicators at National and Regional Level in 15 Countries. Int. J. Qual. Health Care.

[27] WHO Mental Health and Substance Abuse team (2020). Mental Health ATLAS 2020.

[28] Lauriks S., Buster M.C., de Wit M.A., Arah O.A., Klazinga N.S. (2012). Performance Indicators for Public Mental Healthcare: A Systematic International Inventory. BMC Public Health.

[29] Lora A., Lesage A., Pathare S., Levav I. (2017). Information for Mental Health Systems: An Instrument for Policy-Making and System Service Quality. Epidemiol. Psychiatr. Sci..

[30] Mainz J. (2003). Defining and Classifying Clinical Indicators for Quality Improvement. Int. J. Qual. Health Care.

[31] Samartzis L., Talias M.A. (2019). Assessing and Improving the Quality in Mental Health Services. Int. J. Environ. Res. Public Health.

[32] Lora A., Barbato A., Cerati G., Erlicher A., Percudani M. (2012). The Mental Health System in Lombardy, Italy: Access to Services and Patterns of Care. Soc. Psychiatry Psychiatr. Epidemiol..

[33] Corrao G., Soranna D., Merlino L., Monzani E., Viganò C., Lora A. (2015). Do Patterns of Mental Healthcare Predict Treatment Failure in Young People with Schizophrenia? Evidence from an Italian Population-Based Cohort Study. BMJ Open.

[34] Lora A., Monzani E., Ibrahim B., Soranna D., Corrao G. (2016). Routine Quality Care Assessment of Schizophrenic Disorders Using Information Systems. Int. J. Qual. Health Care.

[35] Corrao G., Barbato A., D’Avanzo B., Di Fiandra T., Ferrara L., Gaddini A., Monzio Compagnoni M., Saponaro A., Scondotto S., Tozzi V.D. (2021). Does the Mental Health System Provide Effective Coverage to People with Schizophrenic Disorder? A Self-Controlled Case Series Study in Italy. Soc. Psychiatry Psychiatr. Epidemiol..

[36] Lora A., Monzio Compagnoni M., Allevi L., Barbato A., Carle F., D’avanzo B., Di Fiandra T., Ferrara L., Gaddini A., Leogrande M. (2022). The Quality of Mental Health Care Delivered to Patients with Schizophrenia and Related Disorders in the Italian Mental Health System. The QUADIM Project: A Multi-Regional Italian Investigation Based on Healthcare Utilisation Databases. Epidemiol. Psychiatr. Sci..

[37] Corrao G., Rea F., Di Martino M., Lallo A., Davoli M., De Palma R., Belotti L., Merlino L., Pisanti P., Lispi L. (2019). Effectiveness of Adherence to Recommended Clinical Examinations of Diabetic Patients in Preventing Diabetes-Related Hospitalizations. Int. J. Qual. Health Care J. Int. Soc. Qual. Health Care.

[38] Conferenza Unificata Stato-Regioni (2014). Definizione dei Percorsi di Cura da Attivare nei Dipartimenti di Salute Mentale per i Disturbi Schizofrenici, i Disturbi Dell’umore e i Disturbi Gravi di Personalità.

[39] National Collaborating Centre for Mental Health (UK) (2022). Depression in Adults: Treatment and Management.

[40] Higgins J.P.T., Thompson S.G., Deeks J.J., Altman D.G. (2003). Measuring Inconsistency in Meta-Analyses. BMJ.

[41] Davidson J.R.T. (2010). Major Depressive Disorder Treatment Guidelines in America and Europe. J. Clin. Psychiatry.

[42] Malhi G.S., Bell E., Bassett D., Boyce P., Bryant R., Hopwood M., Lyndon B., Mulder R., Porter R., Singh A.B. (2023). The Management of Depression: The Evidence Speaks for Itself. Br. J. Psychiatry J. Ment. Sci..

[43] Storace F. La Salute Mentale e i Tagli Alla Sanità. Ecco Perché Si Rischia Il Naufragio Di Un Intero Settore—Quotidiano Sanità. https://www.quotidianosanita.it/studi-e-analisi/articolo.php?articolo_id=31547.

[44] Strawbridge R., McCrone P., Ulrichsen A., Zahn R., Eberhard J., Wasserman D., Brambilla P., Schiena G., Hegerl U., Balazs J. (2022). Care Pathways for People with Major Depressive Disorder: A European Brain Council Value of Treatment Study. Eur. Psychiatry.

[45] Petersen T.J. (2006). Enhancing the Efficacy of Antidepressants with Psychotherapy. J. Psychopharmacol. Oxf. Engl..

[46] McHugh R.K., Whitton S.W., Peckham A.D., Welge J.A., Otto M.W. (2013). Patient Preference for Psychological vs. Pharmacological Treatment of Psychiatric Disorders: A Meta-Analytic Review. J. Clin. Psychiatry.

[47] Orsini L.S., O’Connor S.J., Mohwinckel M.T., Marwood L., Pahwa A.S., Bryder M.N., Dong X., Levine S.P. (2022). Observational Study to Characterize Treatment-Resistant Depression in Germany, France and the United Kingdom: Analysis of Real-World Data Collected through a Survey of Healthcare Professionals. Curr. Med. Res. Opin..

[48] Amick H.R., Gartlehner G., Gaynes B.N., Forneris C., Asher G.N., Morgan L.C., Coker-Schwimmer E., Boland E., Lux L.J., Gaylord S. (2015). Comparative Benefits and Harms of Second Generation Antidepressants and Cognitive Behavioral Therapies in Initial Treatment of Major Depressive Disorder: Systematic Review and Meta-Analysis. BMJ.

[49] Karyotaki E., Smit Y., Holdt Henningsen K., Huibers M.J.H., Robays J., de Beurs D., Cuijpers P. (2016). Combining Pharmacotherapy and Psychotherapy or Monotherapy for Major Depression? A Meta-Analysis on the Long-Term Effects. J. Affect. Disord..

[50] Segal Z., Vincent P., Levitt A. (2002). Efficacy of Combined, Sequential and Crossover Psychotherapy and Pharmacotherapy in Improving Outcomes in Depression. J. Psychiatry Neurosci. JPN.

[51] Dj M., El G., Ja R., Tl S., Rl S. (2003). A Randomized Study of Family-Focused Psychoeducation and Pharmacotherapy in the Outpatient Management of Bipolar Disorder. Arch. Gen. Psychiatry.

[52] Teo A.R., Choi H., Valenstein M. (2013). Social Relationships and Depression: Ten-Year Follow-Up from a Nationally Representative Study. PLoS ONE.

[53] Mittelman M.S., Roth D.L., Coon D.W., Haley W.E. (2004). Sustained Benefit of Supportive Intervention for Depressive Symptoms in Caregivers of Patients with Alzheimer’s Disease. Am. J. Psychiatry.

[54] Mittelman M.S., Ferris S.H., Shulman E., Steinberg G., Ambinder A., Mackell J.A., Cohen J. (1995). A Comprehensive Support Program: Effect on Depression in Spouse-Caregivers of AD Patients. Gerontologist.

[55] OECD (2019). Health at a Glance 2019: OECD Indicators.

[56] Chiu C.-C., Liu H.-C., Li W.-H., Tsai S.-Y., Chen C.-C., Kuo C.-J. (2023). Incidence, Risk and Protective Factors for Suicide Mortality among Patients with Major Depressive Disorder. Asian J. Psychiatry.

[57] Nordentoft M., Wahlbeck K., Hällgren J., Westman J., Osby U., Alinaghizadeh H., Gissler M., Laursen T.M. (2013). Excess Mortality, Causes of Death and Life Expectancy in 270,770 Patients with Recent Onset of Mental Disorders in Denmark, Finland and Sweden. PLoS ONE.

[58] Chang C.-K., Hayes R.D., Broadbent M., Fernandes A.C., Lee W., Hotopf M., Stewart R. (2010). All-Cause Mortality among People with Serious Mental Illness (SMI), Substance Use Disorders, and Depressive Disorders in Southeast London: A Cohort Study. BMC Psychiatry.

[59] Grigoriadis S., Robinson G.E. (2007). Gender Issues in Depression. Ann. Clin. Psychiatry Off. J. Am. Acad. Clin. Psychiatr..

[60] Speca A., Pasquini M., Picardi A., Gaetano P., Biondi M. (2001). Gender psychopathological differences in a general psychiatric population. Off. J. Ital. Soc. Psychopathol..

[61] Hyde J.S., Mezulis A.H. (2020). Gender Differences in Depression: Biological, Affective, Cognitive, and Sociocultural Factors. Harv. Rev. Psychiatry.

[62] Sramek J.J., Murphy M.F., Cutler N.R. (2016). Sex Differences in the Psychopharmacological Treatment of Depression. Dialogues Clin. Neurosci..

[63] Kawachi I., Berkman L.F. (2001). Social Ties and Mental Health. J. Urban Health Bull. N. Y. Acad. Med..

[64] Berkman L.F., Glass T., Brissette I., Seeman T.E. (2000). From Social Integration to Health: Durkheim in the New Millennium. Soc. Sci. Med. 1982.

[65] Videbech P., Deleuran A. (2016). The Danish Depression Database. Clin. Epidemiol..

[66] Paris J. (2014). The Mistreatment of Major Depressive Disorder. Can. J. Psychiatry Rev. Can. Psychiatr..

[67] WHO Mental Health and Substance Abuse team (2022). World Mental Health Report: Transforming Mental Health for All.

