# Supplementary material for: Assessment and Monitoring of the Quality of Clinical Pathways in Patients with Depressive Disorders: Results from a Multiregional Italian Investigation on Mental Health Care Quality (the QUADIM Project)
Source: J Clin Med. 2023 May 5;12(9):3297. doi: 10.3390/jcm12093297 (PMC10179491; doi:10.3390/jcm12093297)
Supplement: Supplementary file 1 [file jcm-12-03297-s001.zip › jcm-2357654-supplementary.pdf]

Assessment and Monitoring of the Quality of Clinical Pathways in Patients with Depressive Disorders: Results from a Multiregional Italian Investigation on Mental Health Care Quality (the QUADIM Project)

Matteo Monzio Compagnoni [1,2], Giulia Caggiu [1,2,3,*], Liliana Allevi [3], Angelo Barbato [4], Flavia Carle [2,5], Barbara D'Avanzo [4], Teresa Di Fiandra [6], Lucia Ferrara [7], Andrea Gaddini [8], Cristina Giordani [9], Michele Sanza [10], Alessio Saponaro [11], Salvatore Scondotto [2,12], Valeria D. Tozzi [7], Giovanni Corrao [1,2], Antonio Lora [2,3,13]

1. Unit of Biostatistics, Epidemiology and Public Health, Department of Statistics and Quantitative Methods, University of Milano-Bicocca, 20126 Milan, Italy; matteo.monziocompagnoni@unimib.it (M.M.C.); giovanni.corrao@unimib.it (G.C.)
2. National Centre for Healthcare Research and Pharmacoepidemiology, University of Milano-Bicocca,
20126 Milan, Italy; f.carle@staff.univpm.it (F.C.); salvatore.scondotto@gmail.com (S.S.); a.lora@asst-lecco.it (A.L.)
3. Department of Mental Health and Addiction Services, ASST Lecco, 23900 Lecco, Italy; l.allevi@asst-lecco.it
4. Department of Health Policy, Istituto di Ricerche Farmacologiche Mario Negri IRCCS, 20156 Milano, Italy; angelo.barbato@marionegri.it (A.B.); barbara.davanzo@marionegri.it (B.D.A.)
5. Center of Epidemiology and Biostatistics, Polytechnic University of Marche, 60121 Ancona, Italy
6. Psychologist, Previously General Directorate for Health Prevention, Ministry of Health, 00144 Rome, Italy; terrydifi@gmail.com
7. Centre of Research on Health and Social Care Management, SDA Bocconi School of Management, Bocconi University, 20100 Milan, Italy; lucia.ferrara@unibocconi.it (L.F.); valeria.tozzi@unibocconi.it (V.D.T.)
8. Regional Agency for Public Health, 00198 Rome, Italy; andrea.gaddini@gmail.com
9. Department of Health Planning, Italian Health Ministry, 00144 Rome, Italy; c.giordani@sanita.it
10. Department of Mental Health and Addiction Disorders Forlì-Cesena, AUSL Romagna, 48121 Cesena, Italy; michele.sanza@auslromagna.it
11. General Directorate of Health and Social Policies, 40127 Bologna, Italy; alessio.saponaro@regione.emilia-romagna.it
12. Department of Health Services and Epidemiological Observatory, Regional Health Authority, 90145 Palermo, Italy
13. Consultant for General Directorate for Welfare, 20124 Milan, Italy

* Correspondence: giulia.caggiu@unimib.it; Tel.: +39-0264485859

SUPPLEMENTARY MATERIAL

Supplementary Table S1. Service interventions, treatments and activities delivered by Community Mental Health Centers (CMHCs) and Day Centers (DCs), and their classification in the Italian Mental Health Information System.

Interventions and activities	Mental Health Information system code
**Generic care**	
Psychiatric visit	01
Individual meeting with a professional	03
Consultation	04
Medico-legal assessment	05
Psychological testing	06
Drug administration	11
Meeting with relatives	12
Staff meeting	15
Attendance to day centre	20
Support to daily living activity	24
Network interventions	26
**Psychosocial interventions**	
Individual living skills training	16
Group living skills training	17
Individual socialization	18
Socialization group	19
Expressive, manual and bodywork individual interventions	21
Expressive, manual and bodywork group interventions	22
Vocational training	23
Support for financial, welfare procedures and leisure activities	25
**Psychotherapy**	
Psychological visit	02
Individual psychotherapy	07
Couple psychotherapy	08
Family psychotherapy	09
Group psychotherapy	10
**Psychoeducation**	
Single family psychoeducation	13
Multifamily group psychoeducation	14

Supplementary Table S2. Diagnostic and therapeutic (ICD-9-CM, ICD-10, and ATC) codes used in the current study for drawing records and fields from Healthcare Utilization databases.

DEPRESSION

	**ICD-10 codes (Lombardy)**
Depressive episode	F32.*
Recurrent depressive disorder	F33.*
Dysthymia	F34.1
Other persistent mood [affective] disorders	F34.8
Persistent mood [affective] disorder, unspecified	F34.9
Other recurrent mood [affective] disorders	F38.1
Other specified mood [affective] disorders	F38.8
Unspecified mood [affective] disorder	F39.*
Adjustment disorders	F43.2
	**ICD-9-CM codes (Emilia-Romagna, Lazio and Palermo)**
Major depressive disorder, single episode	296.2
Major depressive disorder, recurrent episode	296.3
Atypical depressive disorder	296.82
Unspecified episodic mood disorder	296.90
Depressive type psychosis	298.0
Dysthymic disorder	300.4
Adjustment disorder with depressed mood	309.0
Prolonged depressive reaction	309.1
Depressive disorder	311.*
**Drugs**	**ATC codes**
Antidepressants	N06A

OUTPATIENT PROCEDURES

	**National procedure codes**
Electrolytes	91.49.2, 90.40.4, 90.37.4, 90.13.3
Electrocardiogram	89.50, 89.52, 89.54
Psychiatric visit	94.12.1, 94.19.1
Psychological interview	94.09
Standardized assessments using tests	94.01.1, 94.08.3, 94.08.4, 94.08.5, 94.08.6
Couple/Family psychotherapy	94.3, 94.42
Group psychotherapy	94.44

Supplementary Table S3. Baseline characteristics of prevalent patients with depressive disorder treated by DMHs of four Italian areas (Lombardy, Emilia Romagna, Sicily, and Lazio Regions) and in the whole sample. Italy, QUADIM-MAP projects, Italy, 2015-2016.

	Lombardy (N=33,222)	Emilia-Romagna (N=21,698)	Sicily (N=6755)	Lazio (N=17,249)	All together (N=78,924)
**Gender**					
Men	11,760 (35.4%)	7096 (32.7%)	2478 (36.7%)	5693 (33.0%)	27,027 (34.2%)
**Age (years)**					
Mean (SD)	51.6 (15.1)	56.5 (15.3)	54.9 (14.2)	53.6 (14.7)	54.1 (14.8)
18-25	1494 (4.5%)	506 (2.3%)	205 (3.0%)	645 (3.7%)	2850 (3.6%)
26-40	6468 (19.5%)	2886 (13.3%)	893 (13.2%)	2550 (14.8%)	12,797 (16.2%)
41-49	7106 (21.4%)	3798 (17.5%)	1139 (16.9%)	3243 (18.8%)	15,286 (19.4%)
≥50	18,154 (54.6%)	14,508 (66.9%)	4518 (66.9%)	10,811 (62.7%)	47,991 (60.8%)
**Education years**					
0-5	14,000 (42.1%)	4449 (20.5%)	2074 (30.7%)	2798 (16.2%)	23,321 (29.5%)
6-8	13,072 (39.3%)	7116 (32.8%)	4199 (62.2%)	6107 (35.4%)	30,494 (38.6%)
9-13	3297 (9.9%)	6051 (27.9%)	0 (0%)	5222 (30.3%)	14,570 (18.5%)
≥14	559 (1.7%)	1557 (7.2%)	479 (7.1%)	1411 (8.2%)	4006 (5.1%)
Missing data	*2294 (6.9%)*	*2525 (11.6%)*	*3 (0.0%)*	*1711 (9.9%)*	*6533 (8.3%)*
**Job condition**					
Employed	17,278 (52.0%)	6665 (30.7%)	1728 (25.5%)	6615 (38.4%)	32,286 (40.9%)
Unemployed	6699 (20.2%)	10,220 (47.1%)	4942 (73.2%)	9166 (53.1%)	31,027 (39.3%)
Invalid	7228 (21.8%)	554 (2.6%)	81 (1.2%)	0 (0%)	7863 (10.0%)
Missing data	*2017 (6.1%)*	*4259 (19.6%)*	*4 (0.1%)*	*1468 (8.5%)*	*7748* (9.8%)
**Family arrangement** §					
Living with family	25,411 (76.5%)	15,658 (72.2%)	1145 (17.0%)	NA	42,214 (68.4%)
Living in community	520 (1.6%)	201 (0.9%)	736 (10.9%)	NA	1457 (2.4%)
Living alone	5387 (16.2%)	3325 (15.3%)	271 (4.0%)	NA	8983 (14.6%)
Missing data	*1904 (5.7%)*	*2514 (11.6%)*	*4603 (68.1%)*	*NA*	*9021* (14.6%)
**Marital status**					
Unmarried	8245 (24.8%)	5312 (24.5%)	1275 (18.9%)	4677 (27.1%)	19,509 (24.7%)
Married	16,783 (50.5%)	9854 (45.4%)	4351 (64.4%)	7947 (46.1%)	38,935 (49.3%)
Separated	2168 (6.5%)	1043 (4.8%)	355 (5.2%)	1306 (7.6%)	4872 (6.2%)
Divorced	1998 (6.0%)	1573 (7.2%)	148 (2.2%)	933 (5.4%)	4652 (5.9%)
Widow/er	2483 (7.5%)	1863 (8.6%)	520 (7.7%)	1019 (5.9%)	5885 (7.5%)
Missing data	*1545 (4.7%)*	*2053 (9.5%)*	*106 (1.6%)*	*1367 (7.9%)*	*5071* (6.4%)
**Clinical status**†					
Optimal	12,897 (38.8%)	8370 (38.6%)	2346 (34.7%)	7575 (43.9%)	31,188 (39.5%)
Good	10,654 (32.1%)	7646 (35.2%)	2431 (36.0%)	4956 (28.7%)	25,687 (32.5%)
Intermediate	6045 (18.2%)	3278 (15.1%)	1161 (17.2%)	2854 (16.6%)	13,338 (16.9%)
High-intermediate	2109 (6.3%)	1333 (6.2%)	502 (7.4%)	1069 (6.2%)	5013 (6.4%)
Poor	1517 (4.6%)	1071 (4.9%)	315 (4.7%)	795 (4.6%)	3698 (4.7%)

§ Information for Lazio Region was not available for this characteristic, which was calculated on the 61,675 remaining patients.

† The clinical status was assessed by the Multisource Comorbidity Score (MCS) according to the hospital admission and the drugs prescribed in the two-year period before the index date. Five categories of clinical status were considered: optimal (score=0), good (1≤score≤5), intermediate (6≤score≤10), high-intermediate (11≤score≤15) and poor (score ≥ 16).

Supplementary Table S4. Baseline characteristics of patients newly engaged in services with depressive disorder treated by DMHs of four Italian areas (Lombardy, Emilia Romagna, Sicily, and Lazio Regions) and in the whole sample. Italy, QUADIM-MAP projects, Italy, 2015-2016.

	Lombardy (N=4770)	Emilia-Romagna (N=3230)	Sicily (N=1439)	Lazio (N=5795)	All together (N=15,234)
**Gender**					
Men	1946 (40.8%)	1207 (37.4%)	597 (41.5%)	2130 (36.8%)	5880 (38.6%)
**Age (years)**					
Mean (SD)	44.9 (11.9)	45.1 (11.8)	48.5 (11.8)	47.0 (12.0)	46.4 (11.8)
18-25	368 (7.7%)	241 (7.5%)	81 (5.6%)	377 (6.5%)	1067 (7.0%)
26-40	1274 (26.7%)	860 (26.6%)	265 (18.4%)	1260 (21.8%)	3659 (24.0%)
41-49	1216 (25.5%)	834 (25.8%)	316 (22.0%)	1346 (23.2%)	3712 (24.4%)
50-65	1912 (40.1%)	1295 (40.1%)	777 (54.0%)	2812 (48.5%)	6796 (44.6%)
**Education years**					
0-5	1559 (32.7%)	181 (5.6%)	279 (19.4%)	464 (8.0%)	2483 (16.3%)
6-8	1902 (39.9%)	1012 (31.3%)	1024 (71.1%)	2151 (37.1%)	6089 (40.0%)
9-13	657 (13.8%)	1109 (34.4%)	0 (0%)	2006 (34.6%)	3772 (24.8%)
≥14	144 (3.0%)	343 (10.6%)	135 (9.4%)	559 (9.7%)	1181 (7.8%)
Missing data	*508 (10.6%)*	*585 (18.1%)*	*1 (0.1%)*	*615 (10.6%)*	*1709 (11.2%)*
**Job condition**					
Employed	1973 (41.4%)	1430 (44.3%)	501 (34.8%)	2741 (47.3%)	6645 (43.6%)
Unemployed	1296 (27.2%)	1022 (31.6%)	925 (64.3%)	2480 (42.8%)	5723 (37.6%)
Invalid	1043 (21.9%)	47 (1.5%)	12 (0.8%)	0 (0%)	1102 (7.2%)
Missing data	*458 (9.6%)*	*731 (22.6%)*	*1 (0.1%)*	*574 (9.9%)*	*1764 (11.6%)*
**Family arrangement** §					
Living with family	3625 (76.0%)	2250 (69.6%)	219 (15.2%)	NA	6094 (69.7%)
Living in community	82 (1.7%)	21 (0.7%)	165 (11.5%)	NA	268 (2.8%)
Living alone	650 (13.6%)	433 (13.4%)	55 (3.8%)	NA	1138 (12.1%)
Missing data	*413 (8.7%)*	*526 (16.3%)*	*1000 (69.5%)*	*NA*	*1939 (20.5%)*
**Marital status**					
Unmarried	1489 (31.2%)	1060 (32.8%)	323 (22.5%)	1843 (31.8%)	4715 (31.0%)
Married	2161 (45.3%)	1232 (38.2%)	901 (62.6%)	2510 (43.3%)	6804 (44.7%)
Separated	320 (6.7%)	145 (4.5%)	94 (6.5%)	476 (8.2%)	1035 (6.8%)
Divorced	278 (5.8%)	243 (7.5%)	37 (2.6%)	331 (5.7%)	889 (5.8%)
Widow/er	143 (3.0%)	88 (2.7%)	55 (3.8%)	153 (2.7%)	439 (2.9%)
Missing data	*379 (7.9%)*	*462 (14.3%)*	*29 (2.0%)*	*482 (8.3%)*	*1352 (8.9%)*
**Clinical status**†					
Optimal	2683 (56.2%)	1910 (59.1%)	691 (48.0%)	3390 (58.5%)	8674 (56.9%)
Good	1426 (29.9%)	948 (29.4%)	507 (35.2%)	1537 (26.5%)	4418 (29.0%)
Intermediate	407 (8.5%)	223 (6.9%)	153 (10.6%)	588 (10.2%)	1371 (9.0%)
High-intermediate	127 (2.7%)	83 (2.6%)	50 (3.5%)	164 (2.8%)	424 (2.8%)
Poor	127 (2.7%)	66 (2.0%)	38 (2.7%)	116 (2.0%)	347 (2.3%)

§ Information for Lazio Region was not available for this characteristic, which was calculated on the 9439 remaining patients.

† The clinical status was assessed by the Multisource Comorbidity Score (MCS) according to the hospital admission and the drugs prescribed in the two-year period before the index date. Five categories of clinical status were considered: optimal (score=0), good (1≤score≤5), intermediate (6≤score≤10), high-intermediate (11≤score≤15) and poor (score ≥ 16).

Supplementary Table S5 Clinical indicators estimated, in the whole sample and according to gender, in the first year of follow-up for prevalent patients with Depressive disorder treated by DMHs of four Italian areas (Lombardy, Emilia Romagna, Sicily, and Lazio Regions). QUADIM-MAP projects, Italy, 2015-2016

		**Whole sample** (n=78,924)	**Male** (n=27,027)	**Female** (n=51,897)	**SMD**
**ACCESSIBILITY AND APPROPRIATENESS OF MENTAL HEALTH CARE**					
1	Patients with at least one outpatient contact in CMHCs or DCs	**92.4%**	92.3%	92.5%	1.3
2	Median number of outpatient contacts in CMHCs *(per PY)*	**5.7**	5.6	5.8	0.0
3	Patients with at least one contact in psychiatric visits	**79.5%**	80.4%	79.1%	4.7
4	Median number of outpatient psychiatric visits *(per PY)*	**3.0**	3.2	3.0	0.0
5	Patients with at least one standardized assessment using tests	**2.5%**	2.8%	2.4%	4.4
6	Median number of standardized assessments using tests *(per PY)*	1.0	1.0	1.0	0.0
7	Patients with at least one home visit §	**4.4%**	4.2%	4.5%	1.8
8	Median number of home visits §	**2.3**	2.0	2.3	0.0
9	Patients treated with at least one psychosocial intervention in CMHCs	**37.5%**	37.0%	37.8%	2.2
10	Median number of psychosocial interventions in CMHCs *(per PY)*	**2.8**	2.5	2.8	0.0
11	Patients treated with at least one psychosocial intervention in CMHCs ψ	**6.8%**	7.5%	6.5%	5.6
12	Median number of psychosocial interventions in CMHCs *(per PY)* ψ	**3.0**	2.9	2.9	0.0
13	Patients treated with at least one psychoeducation session ‡	**1.8%**	2.0%	1.8%	1.8
14	Median number of psychoeducation sessions *(per PY)* ‡	**1. 8**	1.8	1.8	0.0
15	Patients treated with at least one psychotherapy session	**15.6%**	13.5%	16.7%	12.3*
16	Median number of psychotherapy sessions *(per PY)*	**4.8**	4.0	4.8	0.1
17	Patients with at least one outpatient carers' contact	**18.6%**	19.7%	18.0%	6.4
18	Median number of interventions specifically addressed to patients' family members *(per PY)*	**1.8**	1.8	1.8	0.0
19	Patients treated with Antidepressant agents	**65.4%**	61.4%	67.5%	18.2*
20	Patients in both Psychotherapeutic-Pharmacological treatment	**8.4%**	7.1%	9.1%	10.0
21	Patients with at least one admission in residential facilities	**5.5%**	6.4%	5.1%	8.8
22	Median number of days spent in residential facilities *(per PY)*	**89.4**	30.7	88.9	0.5
23	Patients with at least one admission in GHPW	**5.1%**	6.0%	4.7%	8.3
24	Median number of days spent in GHPW *(per PY)*	**14.4**	13.8	14.7	0.0
25	Admissions with a length of stay in GHPW higher than 30 days	**5.7%**	5.1%	6.1%	5.4
26	Unplanned re-admissions in GHPW within 7 days¶	**8.8%**	8.9%	8.7%	0.8

27	Unplanned re-admissions in GHPW within 30 days¶	**17.1%**	16.0%	17.8%	6.8
**CONTINUITY OF MENTAL HEALTH CARE**					
28	Patients with continuous community care	**46.9%**	47.6%	46.6%	2.8
29	Patients persistent with therapy with Antidepressant agents	**51.2%**	49.2%	52.2%	8.1
30	GHPW discharges followed by any mental health outpatient contact within 14 days	**55.0%**	55.9%	54.4%	4.4
31	GHPW discharges followed by an outpatient psychiatric visit within 14 days	**40.4%**	40.5%	40.4%	0.4
32	GHPW discharges followed by home care within 14 days §	**2.9%**	2.3%	3.3%	8.0
**SAFETY OF MENTAL HEALTH CARE**					
33	Patients monitored with electrocardiogram and exam for electrolytes *(in patients treated with antidepressants)*	**19.8%**	20.1%	19.7%	1.4
34	Mortality (SMR), and relative 95% CI	**1.4 (1.1 to 1.2)**	1.5 (1.4 to 1.6)	0.9 (0.8 to 0.9)	10.5*

SMD: Standardized Mean Difference; DMH: Department of Mental Health. CMHC: Community Mental Health Centres; DC: Day-Care Centres; PY: person-year; FGAs: First generation antipsychotics; SGAs: Second generation antipsychotics; GHPW: General Hospital Psychiatric Wards; SMR: standardized mortality ratio.

* Standardized mean differences ≥10% are considered not negligible for differences between males and females distributions.

§ Information for Emilia-Romagna Region was not available for this clinical indicator, which was calculated on the 57,226 remaining patients.

ψ Psychosocial interventions are intended excluding Psychotherapy and Psychoeducation sessions.

‡ Information for Lazio Region was not available for this clinical indicator, which was calculated on the 61,675 remaining patients.

¶ After a previous hospital admission in GHPW (statistical unit)

¥ Values of I^2 for heterogeneity are percentages and can be classified in: Negligible (0-25); Moderate (26-50); Substantive (51-75); Considerable (76-100).

Supplementary Table S6 Clinical indicators estimated, in the whole sample and according to gender, in the first year of follow-up for patients newly engaged in services with Depressive disorder treated by DMHs of four Italian areas (Lombardy, Emilia-Romagna, Sicily, and Lazio Regions). QUADIM-MAP projects, Italy, 2015-2016

		**Whole sample** (n=15,234)	**Male** (n=5880)	**Female** (n=9354)	**SMD**
**ACCESSIBILITY AND APPROPRIATENESS OF MENTAL HEALTH CARE**					
1	Patients with at least one outpatient contact in CMHCs or DCs	**92.3%**	92.0%	92.5%	1.6
2	Median number of outpatient contacts in CMHCs *(per PY)*	**4.5**	4.5	4.3	0.0
3	Patients with at least one contact in psychiatric visits	**75.0%**	77.9%	73.2%	11.1*
4	Median number of outpatient psychiatric visits *(per PY)*	**2.8**	2.5	2.7	0.0
5	Patients with at least one standardized assessment using tests	**4.4%**	5.0%	4.0%	4.6
6	Median number of standardized assessments using tests *(per PY)*	1.0	1.0	1.0	0.0
7	Patients with at least one home visit §	2.2%	2.2%	2.2%	0.2
8	Median number of home visits §	1.6	1.6	1.8	0.0
9	Patients treated with at least one psychosocial intervention in CMHCs	**36.5%**	35.1%	37.4%	4.8
10	Median number of psychosocial interventions in CMHCs *(per PY)*	**2.7**	2.7	3.0	0.0
11	Patients treated with at least one psychosocial intervention in CMHCs ψ	**4.1%**	4.4%	3.9%	2.7
12	Median number of psychosocial interventions in CMHCs *(per PY)* ψ	**2.0**	2.0	1.7	0.0
13	Patients treated with at least one psychoeducation session ‡	**1.8%**	1.9%	1.7%	2.0
14	Median number of psychoeducation sessions *(per PY)* ‡	**1.0**	1.0	1.8	0.1
15	Patients treated with at least one psychotherapy session	**22.0%**	18.4%	24.2%	14.1*
16	Median number of psychotherapy sessions *(per PY)*	**4.0**	3.7	4.2	0.0
17	Patients with at least one outpatient carers' contact	**14.9%**	16.0%	14.2%	4.9
18	Median number of interventions specifically addressed to patients' family members *(per PY)*	**1.7**	1.7	1.5	0.1
19	Patients treated with Antidepressant agents	**43.2%**	43.3%	43.2%	0.3
20	Patients in both Psychotherapeutic-Pharmacological treatment	**8.0%**	7.0%	8.7%	6.1
21	Patients with at least one admission in residential facilities	**3.4%**	4.0%	2.9%	6.1
22	Median number of days spent in residential facilities *(per PY)*	**25.7**	24.3	24.7	0.0
23	Patients with at least one admission in GHPW	**3.3%**	4.3%	2.7%	9.0
24	Median number of days spent in GHPW *(per PY)* 24.3	**13.6**	13.8	13.6	0.0
25	Admissions with a length of stay in GHPW higher than 30 days	**4.9%**	4.4%	5.4%	4.9
26	Unplanned re-admissions in GHPW within 7 days¶	**7.5%**	8.3%	6.6%	6.6
27	Unplanned re-admissions in GHPW within 30 days¶	**29.8%**	30.6%	29.1%	4.6

**CONTINUITY OF MENTAL HEALTH CARE**					
28	Patients with continuous community care	**28.2%**	28.7%	27.9%	1.7
29	Patients persistent with therapy with Antidepressant agents	**34.2%**	31.6%	35.8%	8.8
30	GHPW discharges followed by any mental health outpatient contact within 14 days	**60.6%**	63.9%	57.4%	13.4*
31	GHPW discharges followed by an outpatient psychiatric visit within 14 days	**44.9%**	49.2%	40.7%	17.2*
32	GHPW discharges followed by home care within 14 days §	**1.4%**	1.5%	1.4%	0.5
**SAFETY OF MENTAL HEALTH CARE**					
33	Patients monitored with electrocardiogram and exam for electrolytes *(in patients treated with antidepressants)*	**13.6%**	13.5%	13.6%	0.2
34	Mortality (SMR), and relative 95% CI	**1.1 (1.0 to 1.3)**	1.3 (1.1 to 1.5)	1.0 (0.9 to 1.2)	4.7

SMD: Standardized Mean Difference; DMH: Department of Mental Health. CMHC: Community Mental Health Centres; DC: Day-Care Centres; PY: person-year; FGAs: First generation antipsychotics; SGAs: Second generation antipsychotics; GHPW: General Hospital Psychiatric Wards; SMR: standardized mortality ratio.

* Standardized mean differences ≥10% are considered not negligible for differences between males and females distributions.

§ Information for Emilia-Romagna Region was not available for this clinical indicator, which was calculated on the 12,004 remaining patients.

ψ Psychosocial interventions are intended excluding Psychotherapy and Psychoeducation sessions.

‡ Information for Lazio Region was not available for this clinical indicator, which was calculated on the 9,439 remaining patients.

¶ After a previous hospital admission in GHPW (statistical unit)

¥ Values of I^2 for heterogeneity are percentages and can be classified in: Negligible (0-25); Moderate (26-50); Substantive (51-75); Considerable (76-100).

Acknowledgments. We thank the "QUADIM project" and "Monitoring and assessing diagnostic-therapeutic paths (MAP)" working groups of the Italian Ministry of Health.

"*QUADIM project*" working group (Italian Health Ministry, Prevention Dept):

- Italian Ministry of Health, General Directorate for Health Prevention: Di Fiandra T, Magliocchetti N.;
- Department of Mental Health, Lecco Hospital, Lecco, Italy: Lora A, Allevi L, Barri M.;
- Emilia-Romagna Region: Saponaro A.;
- Lazio Region: Gaddini A, Mattia V.;
- Sicily Region, Regional Health Authority: Scondotto S, Pollina Addario W, Berardi M, Di Giorgi M.;
- University of Milano-Bicocca, Department of Statistics and Quantitative Methods: Corrao G, Monzio Compagnoni M, Caggiu G.;
- IRCCS Mario Negri: Barbato A, D'Avanzo B, Monti I.;
- SDA Cergas Bocconi: Tozzi V.D, Ferrara L, Banks H..

"*Monitoring and assessing diagnostic-therapeutic paths (MAP)*" working group (Italian Health Ministry, Health Planning Dept):

- Italian Ministry of Health, Dept of Health Planning: Office Director, Cristina Giordani (technical coordinator), Maria Donata Bellentani, Carla Ceccolini; Rosanna Mariniello, Modesta Visca; Dept of health prevention: Natalia Magliocchetti, Giovanna Romano; External Expert: Andrea Di Lenarda, Antonio Lora, Paola Pisanti, Rinaldo Zanini;
- Polytechnic University of Marche: Flavia Carle (scientific coordinator), Marica Iommi, Edlira Skrami;
- University of Milano-Bicocca, Laboratory of Healthcare Research and Pharmacoepidemiology: Giovanni Corrao, Federico Rea, Anna Cantarutti, Matteo Monzio Compagnoni, Pietro Pugni;
- Department of Epidemiology Lazio Region: Marina Davoli, Mirko Di Martino, Adele Lallo;
- Aosta Valley Region: Guido Giardini, Patrizia Vittori;
- Campania Region: Alfonso Bernardo, Anna Frusciante;
- Emilia-Romagna Region: Rossana De Palma;
- Friuli-Venezia Giulia Region: Marisa Prezza, Alfredo Perulli;
- Lazio Region: Danilo Fusco, Chiara Marinacci;
- Lombardy Region: Francesco Cideni, Olivia Leoni;
- Marche Region: Marco Pompili, Simone Pizzi;
- Molise Region: Lolita Gallo;
- Puglia Region: Ettore Attolini, Vito Lepore;
- Sicily Region: Salvatore Scondotto, Giovanni De Luca;
- Tuscany Region: Paolo Francesconi, Carla Rizzuti;
- Veneto Region: Francesco Avossa, Silvia Vigna;
- Research and Health Foundation (Fondazione ReS -Ricerca e Salute-): Nello Martini, Antonella Pedrini, Carlo Piccinni, Letizia Dondi.
- National Agency for Regional Health Services: Mimma Cosentino, Maria Grazia Marvulli.
- ANMCO (National Association of Hospital Cardiologists) Study Center: Aldo Maggioni.
